# The end of balloons? Our take on the UK-REBOA trial

**DOI:** 10.1186/s13049-023-01142-5

**Published:** 2023-10-31

**Authors:** Jostein Rødseth Brede, Marius Rehn

**Affiliations:** 1 Department of Emergency Medicine and Pre-Hospital Services, St. Olav’s University Hospital, Prinsesse Kristinas Gate 3, 7006 Trondheim, Norway; 2 Department of Research and Development, Norwegian Air Ambulance Foundation, Oslo, Norway; 3 Department of Anaesthesiology and Intensive Care Medicine, St. Olav’s University Hospital, Trondheim, Norway; 4 Division of Prehospital Services, Air Ambulance Department, Oslo University Hospital, Oslo, Norway; 5 Institute of Clinical Medicine, University of Oslo, Oslo, Norway

**Keywords:** Resuscitative endovascular balloon occlusion of the aorta, REBOA, Aortic occlusion, UK-REBOA trial, Non-compressible haemorrhage

## Abstract

**Background:**

Resuscitative endovascular balloon occlusion of the aorta (REBOA) is increasingly used. The recently published UK-REBOA trial aimed to investigate patients suffering haemorrhagic shock and randomized to standard care alone or REBOA as adjunct to standard care and concludes that REBOA may increase the mortality.

**Main body:**

In this commentary we try to balance the discussion on use of REBOA and address limitations in the UK-REBOA trial that may have influenced the outcome of the study.

**Conclusion:**

The situation is complex, and the patients are in extremis. In summary, we do not think this is the end of balloons.

## Background

Resuscitative endovascular balloon occlusion of the aorta (REBOA) is increasingly used, with haemorrhagic shock as the most common indication [1]. The idea is intuitive, proximal aortic occlusion will limit blood flow to site of injury thereby limiting major haemorrhage. However, REBOA in trauma care is not without controversy, there are believers and non-believers and complications are reported [2, 3]. The authors of this editorial have been involved in REBOA-research in cardiac arrest for years with a subsequent risk for being biased.

Regardless, the recent publication of the UK-REBOA trial [4] warrants balanced discussion. Firstly, the authors should be commended, as the planning, preparation and execution of a clinical randomised controlled trial (RCT) in such a demanding setting is a monumental effort. Conducting trials with randomised design is pivotal to provide more solid evidence for early resuscitation efforts.

## Main text

The UK-REBOA trial aimed to investigate patients suffering haemorrhagic shock and randomized to standard care alone (SC) or REBOA as adjunct to standard care. The study (and subsequently commentators on social media platforms such as Twitter/X) concludes that REBOA may increase the mortality [5], since after 90 days, 54% of the REBOA patients and 42% of the SC patients had died (Odds Ratio 1.58). Other sources with significant first-hand experience with REBOA [6, 7] and a related editorial in JAMA [8] tried to paint a more nuanced picture of the results.

The study leaves numerous points to discuss and to cut it short, we do not necessarily agree with the study’s conclusion.

Just 90 out of the intended 120 patients were included, with 44 patients in the SC group and 46 in the REBOA group. Randomization is a means to obtain matching study groups, but given that the trial was stopped early, the groups may not actually be comparable. A sensitivity analysis was performed to adjust for differences, without significant effects. Still, there are some striking differences between the groups that may not have been included into the analysis.

All patients were critically injured, with a median injury severity score of 41. Twenty-three percent of the patients were in cardiac arrest at some point, demonstrating the severity of the situation and realistically poor prognosis, as cardiac arrest following trauma has devastatingly low survival [9]. The REBOA group had in general lower systolic BP than the SC group making it questionable whether these patients may have survived, regardless of advanced resuscitation.

The abbreviated injury scales in the groups were similar, except for head injury where the REBOA group scored higher. Traumatic brain injury itself is associated with mortality [10]. Further, REBOA may increase blood pressure proximal to the occlusion as demonstrated in both human [11] and animal studies [12–15], carrying that cerebral haemorrhage plus REBOA is likely harmful.

Only 19 (41%) patients received aortic occlusion in the REBOA group and arterial access failed in 8 (17%) patients. Two (5%) patients in the SC group received REBOA, without explanation for the cross-over. Hence, this is a very low number for a strong worded conclusion.

However, this is not the most concerning finding. It’s the matter of minutes. We recognise that this is not the authors fault, but more a systematic health care limitation. Prehospital times were long, with a median of 90 min from injury to hospital arrival. As prehospital physicians, we understand that prehospital time may be prolonged due to weather, difficulties to extract trauma patients etc., but 90 min from injury to admission, without haemorrhage control, will surely influence outcome. The time from randomization in the emergency room to ‘definitive haemorrhagic controlling procedure’ was 64 min in the SC and 83 min in the REBOA group. The interquartile range in the REBOA group was 56 to concerning 156 min. This leaves the patient with hours of bleeding prior to being subject to a haemorrhage controlling procedure. Such time expenditure is neither the study nor the REBOA procedure’s fault, but a characteristic of the health care system investigated.

Further, 32 min to perform REBOA is a surprisingly high procedure time, considered this is performed in the emergency room with adequate ambient lighting and temperature, multiple available personnel and equipment. In the setting of out-of-hospital cardiac arrest, REBOARREST trial participants use approximately 12 min on the REBOA procedure (one physician and one paramedic) [16, 17], which includes the necessary time to unpack the equipment and prepare the patient. Both studies perform the intervention in a low-flow state. A rigorous training, and re-training, program is important to maintain low procedure times. However, a simulated setting will never equal real life. The paper describe that all operators were well trained in the procedure, but the study includes only 19 REBOA balloons distributed upon several hospitals and operators over 4,5 years. This is fewer procedures than some trauma centres perform annually and will potentially limit the learning curve of both operators and teams. It is demonstrated that survival is higher in centres with high vs low REBOA procedure volume [18]. Hence, unsurprisingly no difference was found after the principal stratum analysis for learning curve effect and adds to our perception that this trial demonstrates the real-life challenge to obtain arterial access.

## Conclusions

Obviously, these patients are in extremis and the situation is complex. With all the abovementioned limitations, we believe that it is wrong to solely credit (or discredit) REBOA for the results. To successfully salvage the critically injured patient, we need to improve the sum of marginal gains. Short prehospital time and procedural competence are two obvious factors of importance. In the UK-REBOA trial most patients did not receive a definitive haemorrhagic controlling procedure and exsanguinated. Hence, we think that the UK-REBOA trial does not describe the true effect of REBOA. We still consider REBOA as a potential bridge to therapy, but emphasize that it is important to avoid delay in time to definitive surgery [19–21]. More than half an hour used on the REBOA procedure, after admission to hospital, will likely not benefit the patient. Further studies, with rigorous training and re-training for rapid femoral arterial access and with accurate patient selection should be performed—in high-volume centres.

Additionally, this harmonizes with our comment published earlier [22] where we claim that trauma may neither be the only nor the best indication for REBOA. Many patients suffering major haemorrhage have non-traumatic aetiology and in Norway these constitute the vast majority [23, 24]. REBOA may be beneficial for selected patients suffering cardiac arrest [17] and may be even more beneficial for women with post-partum haemorrhage [22, 25, 26].

In summary, we do not think this is the end of balloons. We salute the investigators for their efforts and challenge others to assess REBOA in non-traumatic haemorrhage.

## Data Availability

Not applicable.

## Abbreviations

REBOA: Resuscitative endovascular balloon occlusion of the aorta
RCT: Randomized controlled trial
SC: Standard care
